# Protecting Family Interests: An Interview Study with Foreign-Born Parents Struggling On in Childhood Cancer Care

**DOI:** 10.1155/2012/681301

**Published:** 2012-03-04

**Authors:** Pernilla Pergert, Solvig Ekblad, Olle Björk, Karin Enskär, Tom Andrews

**Affiliations:** ^1^Childhood Cancer Research Unit, Department of Women's and Children's Health, Karolinska Institutet, Astrid Lindgren Children's Hospital, Q6:05, 171 76 Stockholm, Sweden; ^2^Department for Learning, Informatics, Management and Ethics (LIME), Center for Medical Education (CME), Karolinska Institutet, 171 77 Stockholm, Sweden; ^3^Department of Nursing Science, School of Health Sciences, Jönköping University, P.O. Box 1026, 551 11 Jönköping, Sweden; ^4^The School of Life Sciences, University of Skövde, P.O. Box 408, 541 28 Skövde, Sweden; ^5^School of Nursing and Midwifery, Brookfield Health Science Complex, University College Cork, Cork, Ireland

## Abstract

Sweden's population is gradually changing to become more multiethnic and diverse and that applies also for recipients of health care, including childhood cancer care. A holistic view on the sick child in the context of its family has always been a cornerstone in childhood cancer care in Sweden. The purpose of this study was to gain knowledge about the experiences and main concern of foreign-born parents in the context of paediatric cancer care. Interviews were performed with eleven foreign-born parents and data were analysed using a classic grounded theory approach. Foreign-born parents often feel in a position of powerless dependence, but family interests are protected in their approaches to interaction with healthcare staff, through cooperation, contesting, and reluctant resigning. Healthcare staff need to listen to foreign-born parents and deal with their concerns seriously to prevent powerless-dependence and work for trustful cooperation in the common fight against childhood cancer.

## 1. Introduction

Sweden is gradually changing to a religiously and culturally diverse as well as multiethnic society and the Swedish population includes 19% with a foreign background [1]. According to the Swedish National Board of Health and Welfare people with a foreign background are more likely to be disadvantaged when it comes to socioeconomic situation [2] and health [3]. However, health care is publicly funded and available to everyone.

Childhood cancer care includes very advanced medical, supportive, and nursing care, and in Sweden it is centralized to six childhood cancer care centres. A holistic care of the sick child in the context of its family has always been important in childhood cancer care in Sweden [4] and family-centred care is a pillar in paediatric care [5]. Family-centred care not only includes family involvement in the care of the child but views the family as the recipient of care as well and this should be planned to benefit the whole family [5]. Parents report that the quality of care is of great importance and is reflected in the creation of trust [6]. Furthermore, rapport between parents and staff, particularly nurses, is very important for the interaction to be perceived as positive by parents but also for effective sharing of the care of the child [7]. Parents want to be involved in the care of their child [8] but parental involvement is influenced by support, professionalism, work environment, and responsibility [9]; responsibility in terms of keeping track of and checking the child's treatment [9]. Routines for involving parents in care have been found to be better in paediatric oncology units in Sweden compared to other units at children's hospitals [10] but there are barriers to this involvement that need to be overcome if parents are to be successfully involved in care. Linguistic, cultural, and religious differences as well as organizational obstacles hinder the development of rapport and caring relationships [11] and thus also parental involvement in care. In instances where providers and recipients of health care do not speak the same language, developing an effective caring relationship can be impeded [11] and an increase of the risk of serious medical events in paediatric care has also been found [12].

 In paediatric oncology, parents are expected to be increasingly involved in the care of their child [13] but the experience of foreign-born parents is not well known in this context. The purpose of this study was to gain knowledge about foreign-born parents' main concern and their experiences of the situation, including their child's illness and treatment, and social interactions in the context of paediatric oncology care and to explain how this was dealt with.

## 2. Materials and Methods

In this qualitative study, a classical grounded theory approach [14–16] was chosen to discover how participants deal with their social situation and interactions; resolving their main concern.

### 2.1. Sampling

In accordance with the method [14–16], sampling was guided by the purpose of the study; aiming for easily accessible people with personal experience and knowledge about the subject. The first author and a care coordination nurse identified potential participants, at one paediatric oncology centre, through a list of newly diagnosed patients.

Inclusion criteria were as follows:

- foreign-born parent.
- presence of the parent at the hospital during the child's admissions.
- at least 3 months since the diagnosis.
- the child was still undergoing treatment at the childhood cancer care unit.

A care coordination nurse, who was not involved either in the research project or in the patients' direct care, invited potential participants to participate in the study and three declined. The care coordinator nurse also gave them written information in Swedish or Arabic about the aim and the procedure of the study. Also parents from other language groups were invited to participate and interpreters were offered, however, no written information was available in any other languages. After that the first author contacted parents, who consented verbally to participate, to decide on the date, time, and place for the interview. This resulted in 11 parents (10 mothers, 1 father); demographic data of the participants are presented in Table 1.

### 2.2. Data Collection

Data were collected by means of in-depth individual interviews. They varied in length; 65–120 minutes (median 91 minutes) with interpreter and 30–170 minutes (median 74 minutes) without interpreter. The interviews took the form of semistructured conversations where the first author invited participants to tell about their experiences of their child's illness and treatment. Sociodemographic data were collected by questionnaires available in Swedish and Arabic. An independent authorized translator translated the questionnaire and the interview guide into Arabic, after that an interpreter translated them back to Swedish and the two versions were compared to identify differences. The Swedish language was used in all interviews but interpreters of own mother tongues were offered to all participants. Relating to participants' preferences, all but four of the interviews were conducted without interpreters, with the rest facilitated by the same female Arabic-speaking authorized healthcare interpreter. Triangular seating and consecutive translation were used during the interviews and the interpreter was informed of the aim of the study. Field notes were taken directly after each interview and all were audio recorded and transcribed.

### 2.3. Data Analysis

Field notes and transcribed interviews were coded using the qualitative data analysis software NVivo 2.0 [17] as a tool. Open coding line-by-line was used until the core and related categories had emerged. During coding, questions were asked of the data including “What category does this indicate?,” “What is the main concern of the participants?,” and “How do participants resolve their main concern?” [15]. Codes were grouped together into concepts, which in classical grounded theory is the naming of a pattern of behaviour [14]. Consistent with grounded theory, when the core concept emerged, selective coding was conducted. This was combined with theoretical sampling, thus data was collected, coded, and analysed jointly to enable the process of deciding what data to collect, based on the selected codes, and this modified probing questions used in subsequent interviews [14–16]. The results are about these concepts rather than about persons. The second author validated the codes and concepts by listening to the recordings independently, as well as by reading transcripts and field notes. The emerging concepts were discussed with the first author. Later in the process also literature was used as data in what is termed constant comparison in grounded theory [15]. Further, theoretical coding included the exploration of potential theoretical codes that could integrate concepts, in this study “approaches” is an example of a theoretical code. Memos were written throughout the analysis; these are notes about codes, concepts, and their relationships to each other. Most of the analyses were performed using Swedish but concepts were also named in English.

### 2.4. Ethical Issues

Ethical approval was obtained from the Regional Ethical Review Board. Before the interviews, the voluntary nature of participation and the right to withdraw at any time were emphasized. To protect confidentiality of participants, only limited information is given in connection with quotes.

## 3. Results

**Figure 1:**
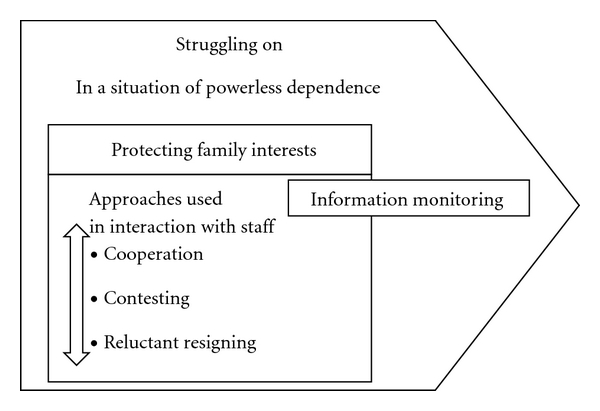
Approaches used by foreign-born parents in interaction with healthcare staff in a situation of powerless dependence.

In the context of childhood cancer care, the main concern of foreign-born parents is to make it through an uncertain situation of extreme emotional burden and stress by *struggling on*. Foreign-born parents often feel in a position of powerless dependence in relation to the child's illness and also in relation to healthcare staff. Protecting family interests is one aspect of struggling on, which encompasses the engagement in information monitoring. Protecting family interests is how parents interact with healthcare staff, which includes cooperation, contesting, and reluctant resigning. The concepts are presented below and are outlined in Figure 1.

### 3.1. Powerless Dependence

Being dependent on others, for care and information, results in perceived powerlessness. The powerlessness is not a matter of complete incapacity but rather a reflection of not being in control.

As parents enter the healthcare system, often with a feeling of being exposed, in need of help and powerless in relation to the child's illness, the interaction with healthcare staff can accentuate or lessen these feelings. Discrimination and suspicion of discrimination accentuate the feeling of powerless-dependence as well as frustration. Furthermore, powerless dependence is influenced by the opportunity for parents to present their point of view and share opinions with healthcare staff and by being listened to. This is influenced by the social status of the parent: “I feel that in Sweden… [people have the attitude toward me that] ‘-You do not speak plain Swedish, you speak with an accent, what do you know?'” (Mother from Europe of a child with leukaemia.)

Additionally, linguistic obstacles negatively influence attempts to present opinions and thereby accentuate the feeling of powerless dependence. The greater the language barrier, the more likely is the feeling of powerless dependence:

I didn't have such a great role [in the treatment decision for my sick child] because I can't speak the language so I couldn't exercise my role (mother from Greater Middle East of a child with brain tumour.)

However, this can be overcome if parents perceive that they are listened to and that their views matter in decisions about the child's care and treatment. Thus, the more the vulnerable person experience that their views matter and that they are listened to, the less the level of felt powerless-dependence.

### 3.2. Information Monitoring

Information monitoring includes health information seeking, avoiding, and controlling behaviours. Information monitoring assists parents in gaining some control over the situation and increasing the possibility that family interests will be protected. The type of information sought includes health-related information about the child's diagnosis, prognosis, the child's present condition such as blood values, and the available treatment options. The latter include treatment alternatives, side-effects, self-help, and seeking a second opinion.

Information is sought from different sources, including physicians, nursing staff, other parents, the sick child, the literature, and the internet. There are different levels of information seeking. For some parents, the level is low because they are already satisfied with, or overwhelmed by, the information at hand, whereas others have a very high level of information seeking and monitoring, as exemplified by “We have received so much information; we have as much as we need… and more to.” (Mother from Europe of a child with a solid tumour.)

You have to ask properly. You have to ask each individual doctor, “what do you think” and “what would you do” and in that way you can get many different opinions and then you have to think… and come up with a joint decision, the best. (Mother from Europe of a child with a solid tumour.)

When satisfied with the information provided by healthcare personnel, parents are less likely to actively seek information. Conversely, disaffection with the information makes it more likely that parents will engage in high level information seeking behaviour to protect family interests.

### 3.3. Protecting Family Interests

The protection of family interests includes protecting parents' own interests and principally those of the sick child. Parents interact with healthcare staff in such a way as to protect family interests and to achieve the best possible care for the child through cooperation, contesting, and reluctant resigning.

Families enter the healthcare system with different levels of trust and with different expectations. Where there is disparity between these expectations and the possibilities of the delivery of care, parents are more likely to actively protect family interests. The approaches used vary with personality, individual preferences, and perceived levels of powerless-dependence. They will also vary for the same parent, who will fluctuate between different approaches, depending on the situation and conditions. All parents use different approaches at different times and can use two approaches simultaneously for different issues or different staff members. Conditions that influence the choice of approaches include the way the family is considered by the staff, attitudes among staff, treatment alternatives, prognosis of the disease, and the family's previous experiences. One example of the latter is experiences of racism. Sometimes these approaches arise as a consequence of interactions with healthcare staff, for example, the establishment of rapport might lead to a trustful cooperation. At other times approaches are deliberate efforts to influence decisions and manoeuvre the care situation and can be considered strategic. Cooperation is the most common approach to protect family interests.

#### 3.3.1. Cooperation

Parents try to stay on good terms with the staff and create good relationships to obtain the best possible care for their child while protecting family interests. One parent said “The moment when I have given my child into … the doctor's hands…I want us together to do the best….” (Mother from Europe of a child with leukaemia.)

Cooperation is negotiated, particularly in relation to the balancing of responsibilities between parents and staff in relation to the care of the child. This negotiation can be open or closed; the latter is achieved with subtle, implicit messages, or even assumptions of role divisions. When subtle messages are misunderstood or not perceived at all, this could lead to conflict in the negotiation. At times, parents entrust responsibilities and decisions to the staff, as exemplified by the following quote: “then it will be fine, if you think that it is the best, then we are on… we say: okay, then we are consenting or what it is called.” (Mother from Europe of a child with leukaemia.)

Cooperation is used by all parents, but the level of trust varies. The higher the level of trust, the better the conditions for cooperation. Trustful cooperation includes acceptance of and confidence in the situation. Cooperation is motivated by the relationship with healthcare staff and by the wish to protect family interests but can also be partly motivated by, or be a consequence of, powerless dependence in an attempt to decrease vulnerability. When trust is lacking or care expectations are not met, cooperation changes to contesting to protect family interests.

#### 3.3.2. Contesting

Contesting is an approach to protect family interests and often includes persuasive communicating, that is, providing convincing information in a persistent way, sometimes with emotional display and threats. Convincing information often concerns needs of the child that parents are trying to convince healthcare staff about, for example, the need to be cared for at a specialist unit or the need for extra recourses or support. Contesting is often linked to a strong feeling of powerless dependence and sometimes also a mistrust of the healthcare staff or healthcare system. Occasionally, contesting is a consequence of a perceived wrongdoing by healthcare staff; at other times, it is a strategic approach to influence and manoeuvre healthcare staff in care decisions. When parents suspect that their interests are being wrongfully ignored, for instance, because of racism and prejudice, they will try to contest for their rights: “I have said outright to the nurses what I think. I said to them—I think that you are unfair between the children.” (Mother from Greater Middle East of a child with leukaemia.)

For many, contesting starts as they try to gain access to care if this is denied, for example, before a serious disease is confirmed, or if they believe that their rights are not being acknowledged:

We were very badly treated actually … sometimes you can see as a foreigner that it is a little bit unfair …Then we came there for the third time in a week… and her father said that “you have to shout at them, you have to tell them that they should examine your daughter properly”. Then I said, “you must come too and say it yourself, you're a man, maybe they will listen.” (Mother from Greater Middle East of a child with leukaemia.)

Contesting is situation dependent and where there is a lack of mutual trust parents are more likely to respond to healthcare staff by contesting. When trusting relations have not been developed, because, for example, of personalities, experience of wrong treatment or obstacles to transcultural caring relationships, parents will try to remain in control by contesting in every possible way to protect family interests: “…you should fight. She [the psychologist] told me to relax and to trust the physicians. … I will control [healthcare staff] as much as possible.” (Mother from Europe of a child with leukaemia.)

Even in trusting relationships, if family interests are threatened in any way, then parents are likely to engage in contesting to achieve their goals. Once goals have been realized, parents attempt to resume trustful cooperation but this can be very difficult to achieve when staff perceive contesting as negative. If this reaction persists, then parents are even more likely to engage in contesting as a response to healthcare staff, thereby creating a causal loop, where parents and staff react to each other in ways that make negative reactions more likely and the reestablishment of trustful cooperation more doubtful.

If parents fail to protect their family interests through a combination of cooperation and contesting, then they are likely to resign themselves to the situation through reluctant resigning.

#### 3.3.3. Reluctant Resigning

Reluctant resigning is an approach to protect family interests and is more likely when parents feel powerless dependence. Parents usually fluctuate between contesting and reluctant resigning. Contesting is used when there is some hope of influencing decisions whereas reluctant resigning is often triggered by a critical juncture, when parents feel unable to influence what is happening or decisions being made without consultation. Simply, they feel disempowered and unable to do anything about it except to accept the situation as it is, or risk permanently alienating staff and becoming exhausted in the process.

Both I and my husband were very irritated by them [physicians]… they tried to persuade us and tell us how we should do this. What could we do?… the only thing that I could say then was this; “if you do not have that experience… this is not a guinea pig who you can just try and try on, so to say, so if there is anywhere else where he could get a better treatment. It's a pity; he's just a small child, to have to expose him to this”. That was the only thing that I could say…I felt like a butchered bird. (Mother from Greater Middle East of a child with brain tumour.)

## 4. Discussion

In this classical grounded theory study with foreign-born parents in paediatric cancer care, struggling on includes information-monitoring such as health information seeking, avoiding and controlling behaviours. Different patterns of information-monitoring (Miller, 1980 in [18]) and information-seeking behaviours [19] have previously been described. It is of great importance to keep parents sufficiently informed, especially because, within paediatric oncology, parents are not currently entirely satisfied with the amount and timing of information [20, 21]. Furthermore, lack of information to parents in paediatric cancer care leads to a feeling of being unwelcome and abandoned [22]. Parents and patients often receive an insufficient level and amount of information, leading to conflict in care [23]. Even though information giving has been a constant subject of nursing research for many years, insufficient information giving continues to be an issue as evidenced in this study. Information monitoring is influenced by psychosocial, cultural, and sociodemographic factors [19], such as ethnicity (Johnson, 1997 in [24]) and it might be expected that there would be a low level of information seeking in this group of foreign-born parents. However, education and social roles also influence information seeking [24] and the findings in the present study that information monitoring is important to protecting family interests might be explained by the fact that participants had a rather high level of education and also by taking into account that they are parents advocating on behalf of their sick children and not themselves.

In a previous study, parents did not view their interactions with nurses as collaborative which is considered the ideal relationship between parents and staff for working together in the care of the child [7]. In the context of the present study, cooperation is an approach for sharing and negotiating the care of the child between parents and staff and thus could form the basis for collaboration.

This study suggests that contextual demands and feelings of powerless dependence of the interviewed parents are the primary reasons for contesting. Failure to attain trust in transcultural caring relationships further hinder the development of such relationships [11]. Also, differences in expectations result in conflicts between parents and healthcare staff in the context of paediatric oncology [23]. Thus, careful inquiry needs to be made into what parents' expectations are and how they can benefit from assistance in assessing the possibilities and realities of the healthcare system [25], to better support and protect them from disappointment and the risk of ending up in reluctant resigning. Contesting might not be an efficient way to protect family interests in care because of the potential risk of alienating staff and the subsequent negative impact on relations and also because healthcare staff prefer trustful cooperation and the development of caring relationships [11, 26].

Persuasive communicating identified in this study is similar to “convincing referral language” used by nurses as they try to communicate their visualization of subtle changes in patients to physicians in a way that is credible [27, 28]. Parents' expressions of anger are often interpreted as a reaction to multiple factors, such as frustration, powerlessness, and suspicions of racism [29]. If contesting includes the expression of negative emotions, it can also have negative consequences for the development of relationships with healthcare staff [29] and has been associated with higher levels of distress in parents [30]. Moreover, Norberg et al. [30] argue that the expression of negative emotions can have negative consequences for communication with healthcare staff.

Language proficiency has previously been identified as a “social marker” to justify the exclusion and negative categorization of people with a foreign accent or nonspeakers of the local language [31]. This experience is also evident in several of the quotes of participants in the present study, where lack of proficiency in the Swedish language becomes an obstacle in protecting family interests in health care. However, one could argue in accordance with Johnstone and Kanitsaki [31] that the problem is the inability of the healthcare system to meet the needs of people lacking proficiency in the local language, categorized as institutional prejudice, leading to discrimination. 

### 4.1. Strengths and Limitations of the Study

Foreign-born parents are often excluded from research because of methodological difficulties related to language differences. Not talking the same language leads to a need to use an interpreter which could be considered a limitation, as findings might be influenced by the interpreter's assumptions and by losing some information in interpretation [32]. However, the voice of foreign-born parents needs to be heard and this is one step from the context of paediatric cancer care in Sweden.

Im et al. [33] suggest five criteria for rigor in cross-cultural nursing research: cultural relevance, contextuality, appropriateness, mutual respect, and flexibility. Even though this is not a cross-cultural study in the sense of comparing different cultures, these criteria are still relevant. The inductive approach of this study ensures several of these criteria are met including cultural relevance—as it aims to study what is of importance to the parents' interviewed; flexibility—to research question; respect—in allowing participants to tell of their own experience. With regard to contextuality, the researchers were well aware of the situation in paediatric cancer care and had a broad understanding of the situation of foreign-born people in Sweden. In the current study, the appropriateness with regard to communication and translation process could be considered fairly good because of the availability of interpreters and the back translation of the written information into Arabic, however, the written information was not made available in any other languages than Arabic and Swedish.

## 5. Conclusions

The approaches identified in this study can provide a better understanding and anticipation of parents' approaches to protecting family interests and healthcare staff can adopt strategies to facilitate cooperation. For example, a more frequent use of interpreters could facilitate trustful cooperation as well as persuasive communicating. However, there are potential difficulties with using interpreters, including the risk of triadic relationships which can hinder the caring relationship, loss of information control and information compacting [11], as well as errors in interpretation [34]. Further research is needed on institutional prejudice and the inability of the healthcare system to meet the needs of people lacking proficiency in the local language. Implementation research would also be desirable as to enable healthcare staff to use available tools and services to bridge obstacles to communication and at the same time gain knowledge of the social processes. Furthermore, healthcare staff need to listen to foreign-born parents and deal with their concerns seriously to prevent powerless-dependence. Healthcare staff also need to properly assess foreign-born parents' expectations of the healthcare system and to further empower them; ensuring that the best interests of their child and family are upheld. Furthermore, it is of great importance to find out what amount and type of information parents want and to give that information in a congruent way. Finally, trust is key to preventing reluctant resigning and for a trustful cooperation in the common fight against childhood cancer.

## Figures and Tables

**Table 1:** Description of the participants.

Number of participants (*n*)	11
Parents' age, range (median)	26–47 (40)

Parental education (*n*)	
Missing data/Less than nine-year compulsory/nine-year compulsory	1/2/2
Upper secondary school/College/University studies	0/5/1

Diagnoses for the informants' children	
Leukaemia/Brain tumour/Solid tumour (both parents of one child).	5/1/4

Parents' country (continent) of origin (*n*)	
Iraq/Syria/Morocco (Greater Middle East)	4/1/1
Finland/Germany/Russia/Serbia Montenegro (Europe)	1/1/1/1
Peru (South America)	1

Parents' mother language (*n*)	
Arabic/Finish/German/Kurdish/Russian/Serbian/Spanish	5/1/1/1/1/1/1

Time interval since immigration, range (median)	2–18 (6)

Self-reported reason for immigration	
Family reunification/Labour market/Refugee /Adventure	8/1/1/1
